# Solvent flashcards: a visualisation tool for sustainable chemistry

**DOI:** 10.1186/s13321-024-00854-9

**Published:** 2024-05-28

**Authors:** Joseph Heeley, Samuel Boobier, Jonathan D. Hirst

**Affiliations:** School of Chemistry, University of Nottingham, University Park, Nottingham, NG7 2RD UK

**Keywords:** Solvent selection, Green chemistry, Solvent flashcards, CHEM21, Visual interface

## Abstract

**Abstract:**

Selecting greener solvents during experiment design is imperative for greener chemistry. While many solvent selection guides are currently used in the pharmaceutical industry, these are often paper-based guides which can make it difficult to identify and compare specific solvents. This work presents a stand-alone version of the solvent flashcards that were developed as part of the AI4Green electronic laboratory notebook. The functionality is an intuitive and interactive interface for the visualisation of data from CHEM21, a pharmaceutical solvent selection guide that categorises solvents according to “greenness”. This open-source software is written in Python, JavaScript, HTML and CSS and allows users to directly contrast and compare specific solvents by generating colour-coded flashcards. It can be installed locally using pip, or alternatively the source code is available on GitHub: https://github.com/AI4Green/solvent_flashcards. The documentation can also be found on GitHub or on the corresponding Python Package Index webpage: https://pypi.org/project/solvent-guide/.

**Scientific Contribution:**

This simple and easy-to-use digital tool provides a visualisation of solvent greenness data through a novel intuitive interface and encourages green chemistry. It offers numerous advantages over traditional solvent selection guides, allowing users to directly customise the solvent list and generate side-by-side comparisons of only the most important solvents. The release as a standalone package will maximise the benefit of this software.

**Graphical Abstract:**

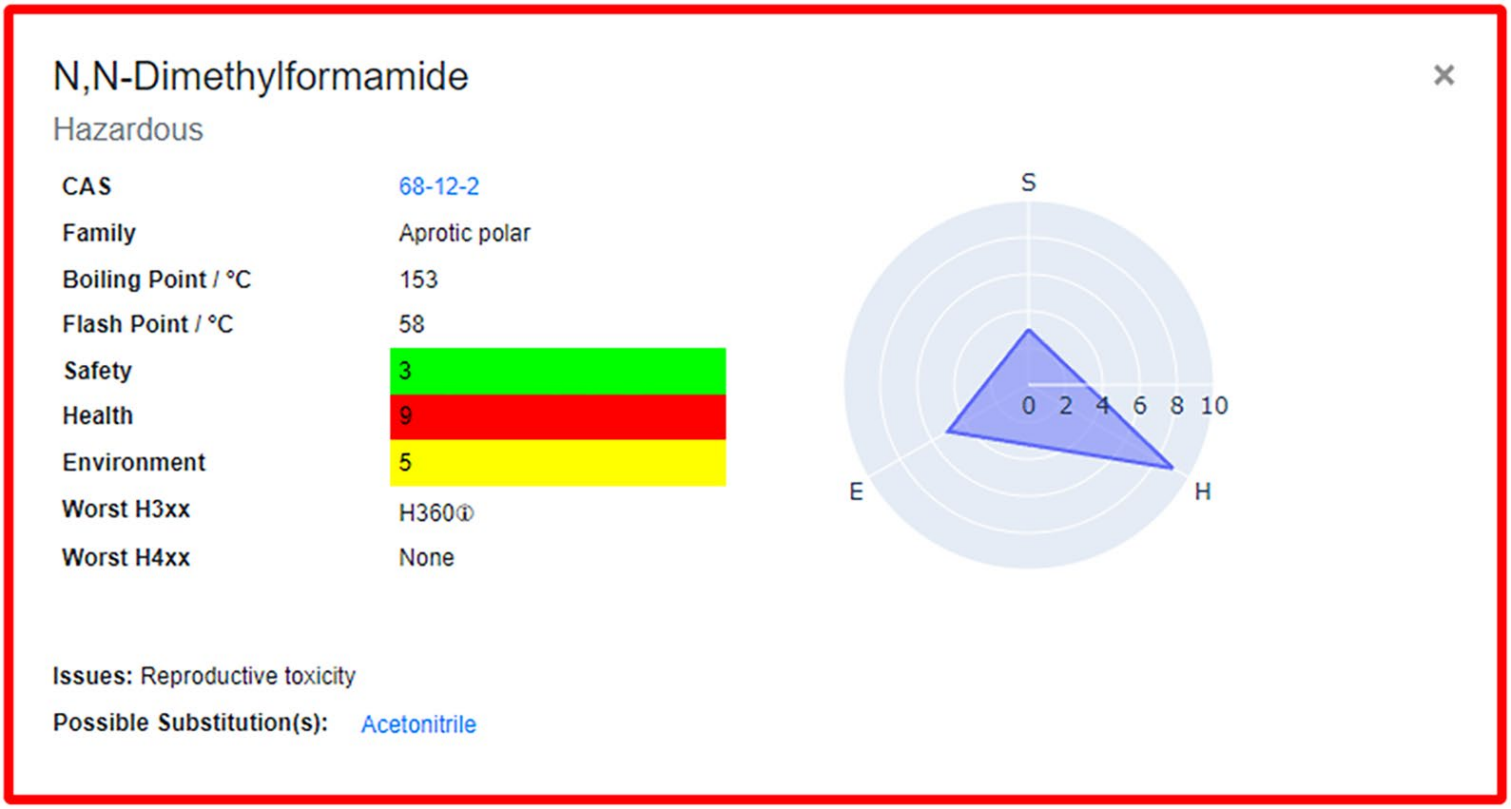

## Introduction

To create greener pharmaceutical processes, greener choices must be made during chemical discovery [1–3]. Making such choices early in the discovery process can lead to easier development at later stages and forms the focus of major industrial initiatives [4]. Digital chemistry tools that can augment decision-making at the laboratory level are thus desirable to allow chemists to rapidly identify and critically evaluate these choices [3, 5].

**Fig. 1:**
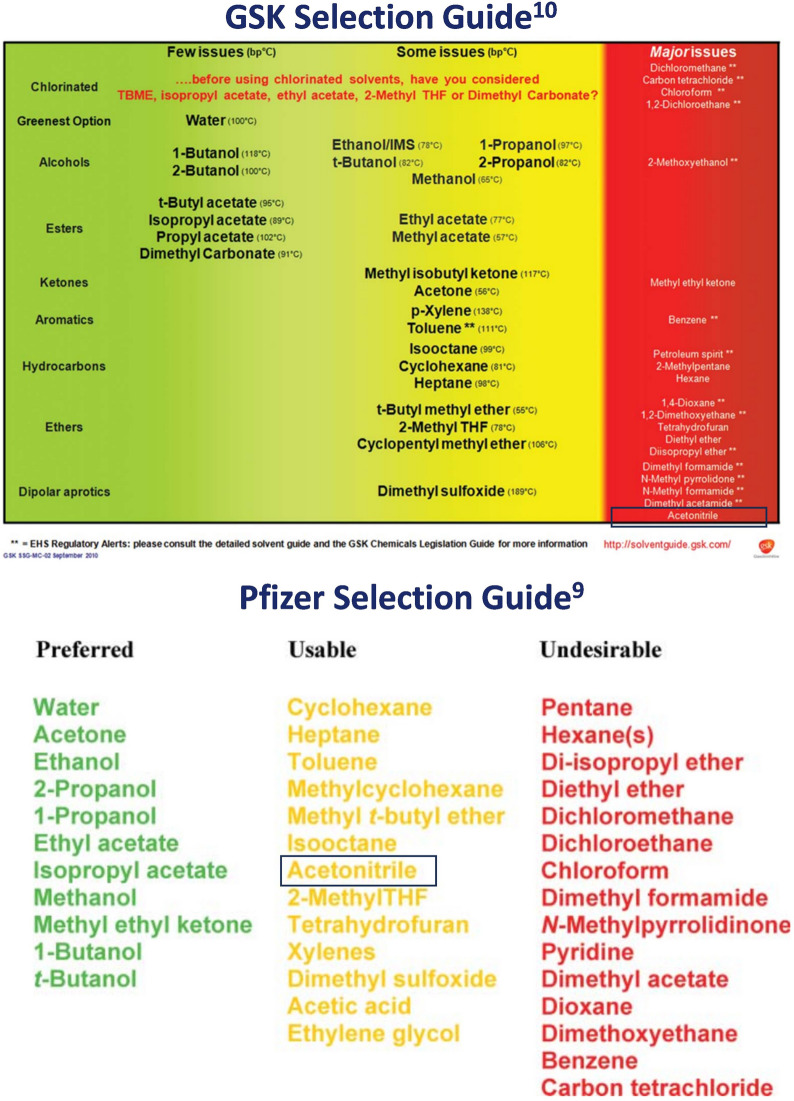
GSK (top) and Pfizer (bottom) solvent selection guides. The position of acetonitrile on both guides has been highlighted. A comprehensive review of available solvent guides has been previously published [7]

Many common lab solvents carry significant environmental hazards and can account for more than half of the waste produced in some pharmaceutical processes [6], making traditional synthetic chemistry inherently polluting. To combat this, many pharmaceutical companies have developed selection guides that visually depict the environmental impact of a range of solvents [7–11]. These guides rank common laboratory solvents using different Safety, Health and Environment (SHE) criteria, giving simple visualisations. While these guides broadly show good agreement with one another [12], there are some conflicting examples: the GSK guide [10] categorises acetonitrile as a solvent with major issues, while the Pfizer [9] and Sanofi [11] guides categorise the solvent as “Usable” and “Recommended”, respectively (Fig. 1). Other common solvents such as 2-methyl tetrahydrofuran and *t*-butanol also show significant disagreement.

To address these issues, the Innovative Medicines Initiative (IMI)-CHEM21 partnership consolidated these data to produce a more holistic solvent selection guide [13]. Each solvent is given three scores of 1–10 that correspond to its impact upon Safety, Health, and the Environment, with higher scores denoting more significant issues. These scores are then combined and discussed to group the solvents into one of four categories: Recommended (green), Problematic (yellow), Hazardous (red) and Highly Hazardous (brown). This methodology was applied to a collection of 53 common laboratory solvents that were included in the original selection guides. This list is a combination of common and less-common laboratory solvents and can be expanded using the spreadsheet tool that was published as part of the supporting information for the original study [13]. It should be noted that this selection guide was designed for use in the pharmaceutical sector, and so CHEM21 SHE scores and classifications may require modification for other applications. For example, for cases where evaporation or distillation is integral to the process, one would not wish to bias against high boiling point solvents.

AI4Green [14] is an open-source electronic laboratory notebook that promotes green chemistry by encouraging users to make greener choices during reaction design. To identify these choices during solvent selection, a simple user interface was developed to visualise the data from the CHEM21 solvent selection guide by generating a series of solvent flashcards that make use of the colour-coding system for easy comparison of solvents. However, as this was built as part of AI4Green, users are required to log in to gain access. It was envisaged that a stand-alone version of this functionality would be useful for researchers who are not currently part of the AI4Green userbase but still require a fast and easy way to compare and identify green solvents. The modular design of the AI4Green code base makes it easy to extract such functionality.

## Implementation

The stand-alone package is written in Python, JavaScript, HTML and CSS and runs a local instance of a Flask application that can be opened in a web browser. The use of the Flask blueprint framework makes integration into other Flask applications easier, so the functionality presented here can be built into other packages and workflows. The application can be downloaded via pip or alternatively the source code can be found on GitHub [15].

**Fig. 2:**
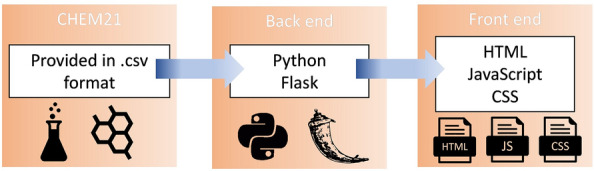
Simple schematic of code structure

A simple schematic of the code structure is shown in Fig. 2. The data from the CHEM21 selection guide is provided as a.csv file which is downloaded during installation and contains all the information needed to generate the solvent flashcards. The backend is built in python and exploits the well-documented Flask framework to run the application. The use of python makes it easy for developers to build additional features, while the use of the Flask blueprint structure makes the application modular and easy to incorporate into other Flask applications. The front end is built in HTML and exploits JavaScript and CSS to dynamically display the CHEM21 data and facilitate user interaction. A Google Colaboratory notebook [16] that runs a demo of this software is provided in the supporting information.

## Discussion

**Fig. 3:**
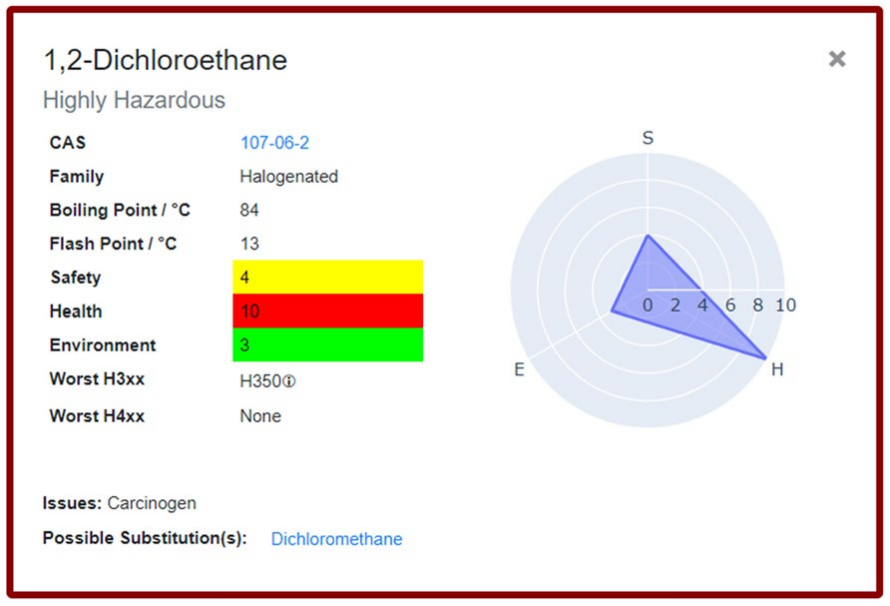
The solvent flashcard generated for 1,2-dichloroethane. The brown outline corresponds to the CHEM21 ranking: Highly Hazardous

Upon launching the application, the user is presented with 53 solvents which are categorised according to functional group (alcohols, halogenated, etc.). These solvents are those that were included as part of the original CHEM21 selection guide, and selecting a solvent generates a corresponding flashcard (Fig. 3). The solvent’s name is shown at the top of the flashcard, followed by its overall CHEM21 ranking which is reflected in the colour-coded border. The CAS number underneath provides a link to the solvent’s entry on the PubChem webpage [17] and the Family details the functional group category of the solvent. The boiling point and flash point are important considerations for preventing explosion during practical chemistry, so these are also provided. The SHE scores from CHEM21 are provided as colour-coded blocks, giving users a full breakdown of the overall CHEM21 ranking. These scores are also presented as a radar plot on the right-hand side, making it quick and easy for users to assess SHE scores and identify areas of concern with a chosen solvent. The most severe health and environmental hazards were also included as part of the CHEM21 selection guide by identifying the hazard codes that contributed most to the SHE scores. These are provided at the bottom of the flashcard as the “Worst H3XX” and “Worst H4XX” categories, and correspond to the Globally Harmonized System (GHS) hazard codes [18]. The associated hazard statements can be viewed by hovering over the information icon. It should be noted that other hazards may be associated the solvent on the flash card. Significant issues that should prevent the use of the solvent and any potential substitutions are given at the bottom of the card. These were taken from the Pfizer solvent selection guide [9] and provide the user with alternatives for any particularly dangerous solvents. These suggestions are intended as generic replacements for specific solvents and are not specific for any individual application. The flashcards can be collapsed by clicking the “x” icon in the top right corner.

**Fig. 4:**
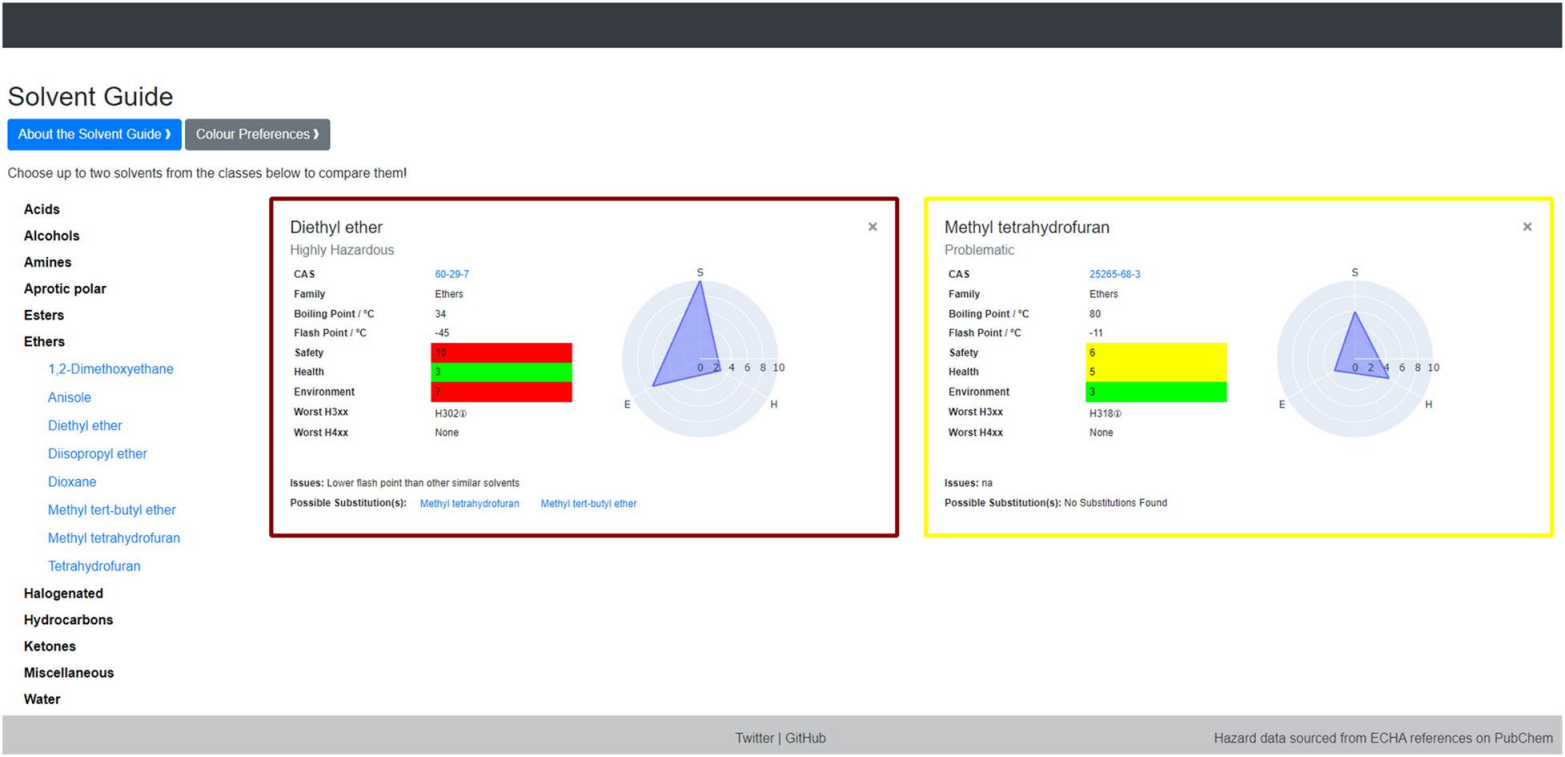
Full example of solvent flashcards showing side-by-side comparison. Families of solvents (shown on left) are expandable, here the “Ethers” family is expanded

**Fig. 5:**
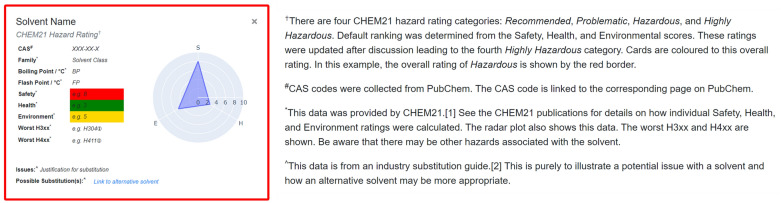
Example solvent flashcard (left) and accompanying explanation of data (right)

The visual interface allows the user to view up to two solvent flashcards at a time, facilitating easy side-by-side comparison. The colour-coding conveys the overall CHEM21 score while the radar plots and colour-coded SHE scores give more in-depth insight when comparing solvents. Enabling users to select and compare individual solvents offers an advantage over the traditional static selection guides, as it allows consideration of only the most relevant data. An example of the full interface, including two flashcards and the list of solvent families, is shown in Fig. 4. The “About the Solvent Guide” button in the top left of the interface takes the user to an information page that shows an example flashcard and gives a full explanation of the data included (Fig. 5). The “Colour Preferences” button allows the user to change the default colours, thereby enhancing accessibility.

**Fig. 6:**
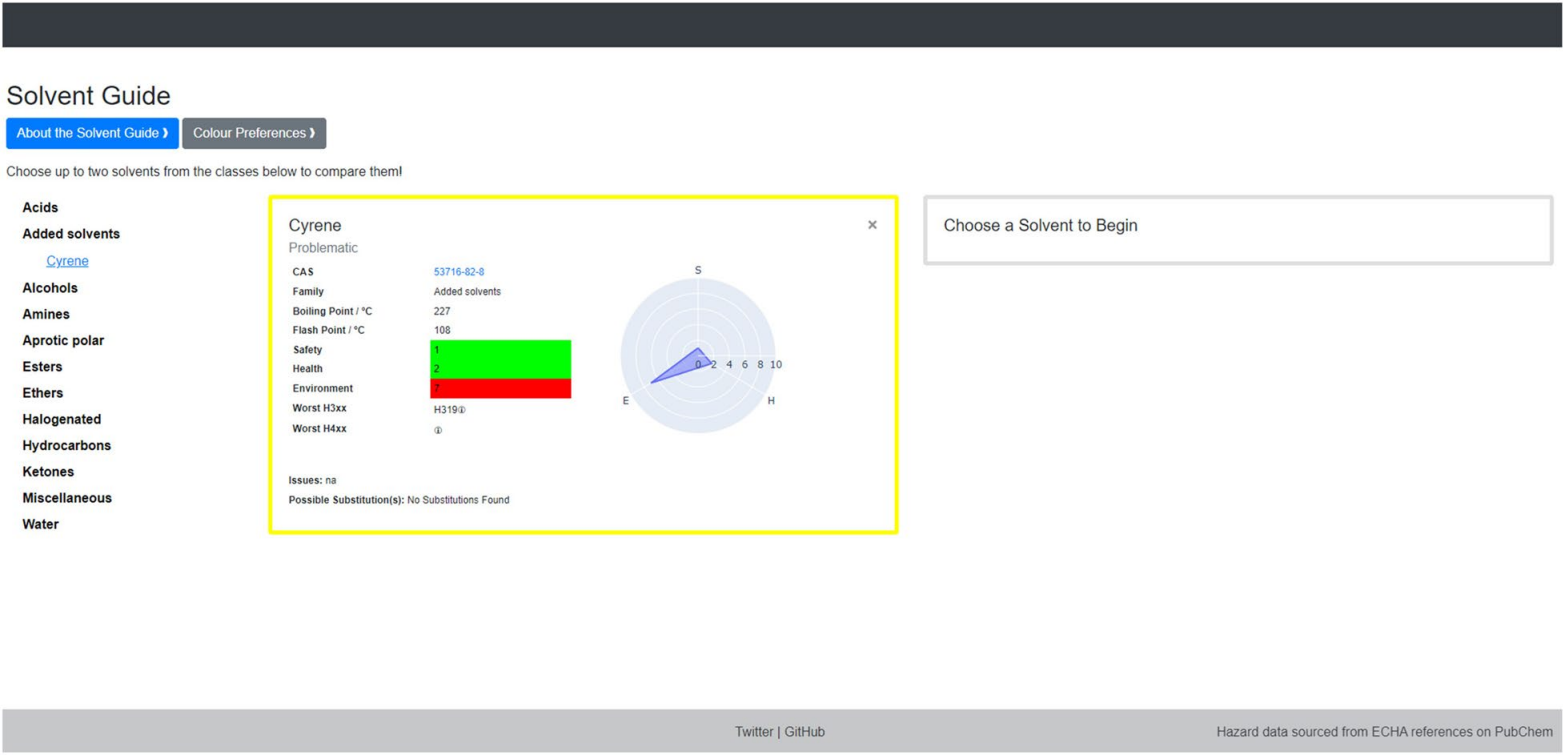
Example of added flashcard for Cyrene. This flashcard has been added to the “Added Solvents” family

As demonstrated by the variability in previous published selection guides, different institutions have different requirements and regulations for solvent selection, making the need to customise the solvent list essential. Solvents can be added using simple in-built functionality, prompting the user to input the information needed to generate a new flashcard which is then saved to the dataset. An example is shown in Fig. 6, where a new flashcard for the solvent Cyrene has been generated and added to a new family (Added Solvents). Flashcards can also be added to existing families. Flashcards can also be removed from the solvent list, which would be useful in cases where banned solvents are contained in the original 53 CHEM21 solvents. The ability to add and remove solvents allows users to tailor the solvent list to reflect their specific usage and needs, resulting in a more dynamic and customisable solvent selection tool.

## Summary

This simple and customisable tool visualises CHEM21 solvent data and provides an easy way to compare solvents interactively by the generation of flashcards. The flashcards present a colour-coded summary of the safety, health, and environmental features of solvents from the CHEM21 selection guide, allowing quick and easy visual comparison of green solvents. These flashcards were originally developed as an integrated part of the AI4Green ELN. To facilitate their usage by a wider community, we have exploited the modular nature of the AI4Green codebase to extract this functionality and package it into a stand-alone version which can be readily incorporated into other workflows and packages. We envisage that these flashcards will be useful to aid solvent selection in pharmaceutical laboratories.

To improve the package further, it would be beneficial to develop a user interface for the addition and removal of solvents. The current method is limited to the command line interface which can be challenging to use. It would also be of use to develop a workflow to calculate the CHEM21 SHE scores for newly added solvents; at present users are required to calculate these scores separately and input them during solvent addition which could provide a barrier to use. Work to address these problems is currently underway.

## Data Availability

The software and dataset are open-source and available for public use under the GNU Affero General Public License version 3.0. Project name: Solvent Guide; Project Homepage: https://github.com/AI4Green/solvent_flashcards; Installation Instructions: can be found at: https://github.com/AI4Green/solvent_flashcards/blob/master/README.md or https://pypi.org/project/solvent-guide/; Operating Systems: Platform independent; Programming Language: Python, HTML, JavaScript, CSS; Other Requirements: dependencies are listed with installation instructions; License: GNU Affero General Public License v 3.0; Data: Included with package on download or can be found online in the source repository: https://github.com/AI4Green/solvent_flashcards/blob/master/src/solvent_guide/sources/blueprints/solvent_guide/CHEM21_full.csv
